# Association Between Season, Temperature and Causative Organism in Microbial Keratitis in the UK

**DOI:** 10.1097/ICO.0000000000001748

**Published:** 2018-09-18

**Authors:** Andrew Walkden, Catherine Fullwood, Shi Zhuan Tan, Leon Au, Malcolm Armstrong, Arun K. Brahma, Jaya D. Chidambaram, Fiona Carley

**Affiliations:** * Cornea Department, Manchester Royal Eye Hospital, Manchester, United Kingdom;; † Centre for Biostatistics, Division of Population Health, Health Services Research and Primary Care, School of Health Sciences, Faculty of Biology, Medicine and Health, University of Manchester, Manchester, United Kingdom;; ‡ Research and Innovation Department, Manchester University NHS Foundation Trust Manchester, United Kingdom;; § Microbiology Department, Manchester Royal Infirmary, Manchester, United Kingdom;; ¶ Clinical Research Department, Faculty of Infectious and Tropical Diseases, London School of Hygiene and Tropical Medicine, London, United Kingdom; and; ‖ School of Medical Sciences, University of Manchester, Manchester, United Kingdom.

**Keywords:** microbial keratitis, corneal ulcer, cornea, season, temperature, acanthamoeba

## Abstract

**Purpose:**

Microbial keratitis (MK) is a major cause of corneal blindness worldwide. Variations in season and temperature can affect MK incidence due to specific causative organisms; however, few studies have examined these factors in the UK.

**Methods:**

Retrospective review of all corneal scrapes from patients with MK presenting to Manchester Royal Eye Hospital, UK, between January 2004 and December 2015. Manchester’s monthly temperature data were obtained from Met Office UK. Analysis was performed using logistic regression.

**Results:**

From 4229 corneal scrapes, 1539 organisms grew (90.6% bacteria, 7.1% fungi, and 2.3% *Acanthamoebae* sp.). Gram-positive bacteria grew with increasing temperature [odds ratio (OR) 1.62, 95% CI: 1.11–2.39, *P* = 0.014], and fungi grew with decreasing temperature (OR 0.29, 95% CI: 0.16–0.51, *P* < 0.001). *Moraxella* sp. grew with decreasing temperature (OR 0.91, 95% CI: 0.86–0.96, *P* = 0.001). Compared with winter, overall culture positivity was significantly less likely in summer (OR 0.57, 95% CI: 0.38–0.87, *P* = 0.008) and spring (OR 0.65, 95% CI: 0.43–0.99, *P* = 0.045). Gram-negative bacteria were more likely in summer (OR 1.48, 95% CI: 1.06–2.09, *P* = 0.022) and autumn (OR 1.75, 95% CI: 1.24–2.47, *P* = 0.001). *Candida* sp. were less likely in summer (OR 0.25, 95% CI: 0.07–0.82, *P* = 0.027) and autumn (OR 0.18, 95% CI: 0.05–0.62, *P* = 0.009), and *Acanthamoeba* sp. were less likely in summer (OR 0.39, 95% CI: 0.15–0.92, *P* = 0.037) and spring (OR 0.26, 95% CI: 0.08–0.69, *P* = 0.011).

**Conclusions:**

Herein we report variation in the incidence of MK-causing organisms by season and temperature; this finding may aid clinicians in predicting possible causative organisms for MK at differing times of the year.

Microbial keratitis (MK), characterized by stromal infiltration with an overlying epithelial defect and evidence of suppuration, is a potentially vision-threatening condition that requires prompt medical attention. If left to progress, MK may cause significant corneal scarring or perforation, and hence empirical treatment may need to be initiated rapidly while awaiting microbiological confirmation of the causative organism. Corneal infections are uncommon unless predisposing factors are present, with contact lens wear being implicated as a major risk factor.^1–4^

In certain geographical locations, microbiological analysis of corneal samples may not be possible. Additionally, some organisms, for example fungi and *Acanthamoeba* sp., may take several days to grow in culture, and as such significant time can elapse before microbiological guidance becomes available. Local epidemiological patterns of infection may, therefore, provide valuable information to guide management. Other authors have documented geographical and seasonal variation amongst causative organisms in MK,^5–7^ but very few studies have explored these factors in the UK.^8,9^ The 2 UK studies that have previously investigated seasonal variation in MK have observed a peak of MK presenting during the summer months (June–August) in both Nottinghamshire and Hampshire in the UK, although variation in the prevalence of causative organisms by season or temperature was not reported.^8,10^

We recently reported temporal trends in the causative organism of MK in patients presenting to Manchester Royal Eye Hospital, UK, over a 12-year period; we observed a decreasing prevalence of gram-positive bacteria over this time period but increasing levels of *Moraxella* sp., *Acanthamoeba* sp. and fungal species in more recent years.^11^ In the present study, we have explored the association between the causative organism, season, and monthly average temperature over the same 12-year time period.

## METHODS

This study was conducted in accordance with the Declaration of Helsinki, and ethical approval was obtained from the National Health Service Research Ethics Committees, UK, via the National Integrated Research Application System, UK. Data on culture positivity, organism cultured, and date of corneal scraping were collected retrospectively for all corneal scrape samples received by the Microbiology Department at Manchester Royal Infirmary from January 1, 2004 to December 31, 2015; microbiological methods were previously described by Tan et al.^11^ An ophthalmology resident, fellow or consultant performed corneal scraping on ulcers with stromal infiltrate measuring ≥1 mm in diameter. The unit of analysis was the corneal scrape; repeated scrapes from the same patient were included if the culture was positive, and multiple organisms from the same scrape were included.

Average monthly temperature data from January 2004 to January 2016 were purchased from the Met Office, Devon, UK (www.metoffice.gov.uk). These readings were taken from the geographical weather stations nearest to Manchester Royal Eye Hospital/Manchester Hulme Library from January 2004 to October 2013 (NGR 3834E 3967N) and Rostherne from November 2013 to January 2016 (3747E 3849N). To examine temporal trends, the study period was divided into groups of 1 year (1st December–30th November of the following year). Seasons were defined as per meteorological definitions that have been used in previous studies,^12^ winter (December 1–February 29 of the following year), spring (March 1–May 31), summer (June 1–August 31), and autumn (September 1–November 30) for each 1-year period. Statistical analysis was performed using R version 3.2.4 (www.R-project.org) with the Analysis of Overdispersed Data package (“aod”).^13,14^ Logistic regression analyses were performed with overall culture positivity as the dependent variable (or culture-positive to specific organisms) and season, year and temperature as independent variables. For season, winter was used as the baseline, and each season was compared with winter (winter was chosen as the baseline as there was more distinction in temperature between this period and temperature in the other seasons). To account for changes over time, an interaction term was considered between season and year that reflects the changing differences between seasons over time. Potential nonlinear relationships were explored by including quadratic and cube terms for both year and temperature. Therefore, some relationships are presented with up to 3 components, each representing a different shape of the relationship. Backward stepwise selection was used to identify the final model with variable selection based on Akaike's Information Criteria. When season was included in the final model, the Wald test was used to assess the overall significance of season.

## RESULTS

**FIGURE 1.**
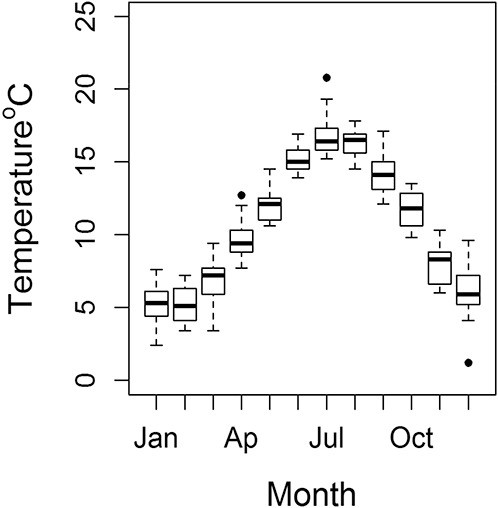
Variation in average monthly temperature during the entire study period, shown in box-and-whiskers plot form.

During the study period, 4229 corneal scrape samples were received, 32.6% of which were culture-positive for bacteria, fungi, or *Acanthamoeba* sp. (a total of 1539 organisms), including 148 mixed infections (ie growth of up to 4 organisms).^11^ The average monthly temperature variation during the entire study period is shown in Figure 1, with a minimum temperature of 1.2°C in December 2010 and a maximum of 20.8°C in July 2006. Exploratory analysis showed no evidence to suggest a systematic increase in average monthly temperature over the 12-year study period (ie temperature–year interaction). Regression results of the final models with no interaction terms are shown in Table 1. Table 2 reports the results of these models with a significant interaction term.

**TABLE 1.**
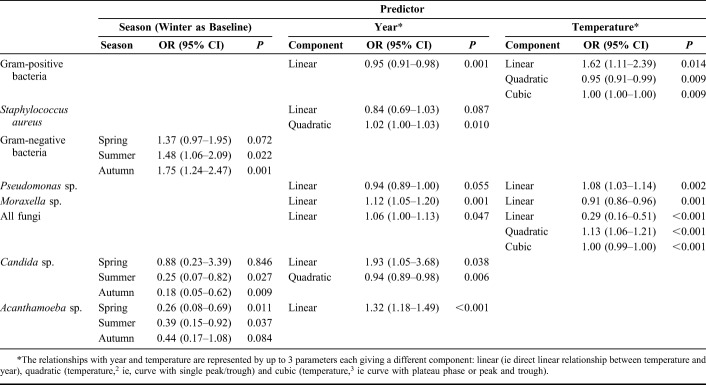
Results of Final Multivariable Regression Models, Without Interaction Term (OR)

**TABLE 2.**
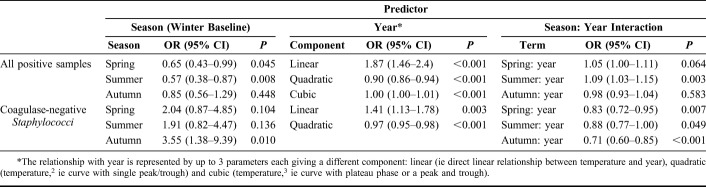
Results of Final Multivariable Regression Models With Interaction Term (OR)

The largest number of culture-positive samples for any organism occurred in the winter season, with culture-positivity less likely in the corneal scrape samples obtained in summer [odds ratio (OR) 0.57, 95% CI: 0.38–0.87, *P* = 0.008], or spring (OR 0.65, 95% CI: 0.43–0.99, *P* = 0.045) compared with winter (Table 1). A temporal interaction was found with a slight increase in the proportion of culture-positive samples occurring in summer rather than winter (OR 1.09, 95% CI: 1.03–1.15, *P* = 0.003; Table 2) with increasing years in the study period.

### Associations Between Specific Organisms, Season and Temperature

**FIGURE 2.**
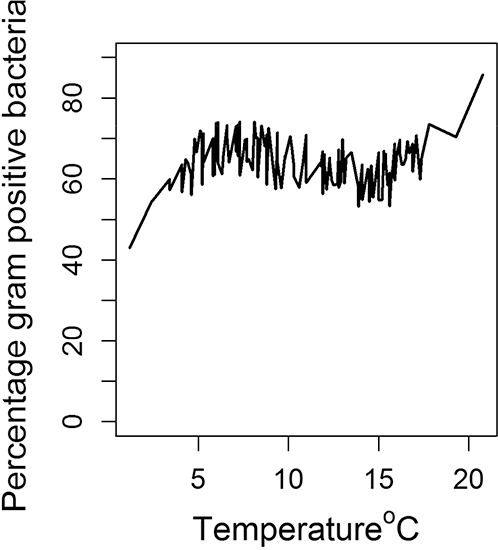
Proportion of gram-positive bacteria cultured as a percentage of all culture-positive organisms, versus temperature.

There was a slight decrease in the isolation of gram-positive organisms over the 12-year period (OR 0.95, 95% CI: 0.91–0.98, *P* = 0.001; Table 1), however there was an increased likelihood of culture-positivity for gram-positive organisms with increasing temperature (linear component OR 1.62, 95% CI: 1.11–2.39, *P* = 0.014; Table 1), with a non-linear relationship, roughly plateauing between 5°C and 15°C (ie cubic relationship) as shown in Figure 2. Amongst the gram-positive organisms, coagulase-negative *Staphylococcus* sp. were the most abundant (34.1%, n = 332/974) and, when compared to all other culture-positives, occurred more frequently in autumn (OR 3.55 95% CI: 1.38–9.39, *P* = 0.010; Table 2).

Gram-negative organisms (n = 420) were more likely to be cultured in summer (OR 1.48 95% CI: 1.06–2.09, *P* = 0.022) or autumn (OR 1.75 95% CI: 1.24–2.47, *P* = 0.001; Table 1) when compared with the winter season. *Pseudomonas* sp. were the most frequently isolated gram-negative organisms (37.1%, n = 156/420), of which 86.0% were *Pseudomonas aeruginosa* (n = 134/156). Increasing temperature was found to be associated with slightly higher odds of culture-positivity for *Pseudomonas* sp. (OR 1.08, 95% CI: 1.03–1.14, *P* = 0.002), but lower odds of culture-positivity for *Moraxella* sp. (n = 93; OR 0.91, 95% CI: 0.86–0.96, *P* = 0.001; Table 1) when compared with all other gram-negative organisms.

**FIGURE 3.**
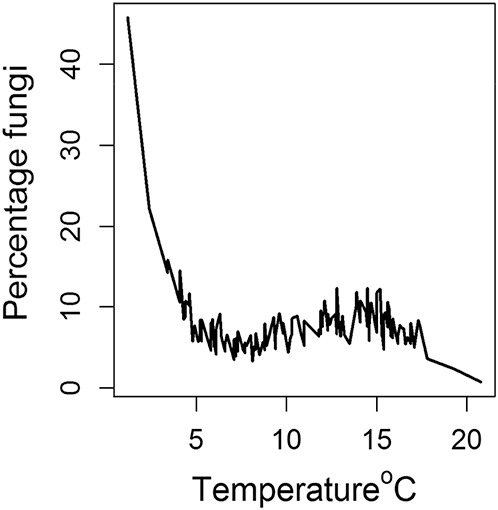
Proportion of fungi cultured as a percentage of all culture-positive organisms, versus temperature.

With regard to fungi, 2.5% of all samples (n = 106) were culture-positive, of which 53.2% grew *Candida* sp. (n = 58/106) and 25.7% grew *Fusarium* sp. (n = 27/106). Overall, there was an increased likelihood of culture-negativity for any fungus with decreasing temperature (linear component OR 0.29, 95% CI: 0.16–0.51, *P* < 0.001; Table 1) roughly plateauing between 5°C and 16°C, as shown in Figure 3. Positive *Candida* samples were less likely in summer (OR 0.25, 95% CI: 0.07–0.82, *P* = 0.027) or autumn (OR 0.18, 95% CI: 0.05–0.62, *P* = 0.009) compared to winter. There was a slight change in the temporal trend with more fungal culture-positivity near to the end of the study (OR 1.06, 95% CI: 1.00–1.13, *P* = 0.047; Table 2).

A temporal trend was also noted for *Acanthamoeba* sp., with more positive cultures from samples obtained toward the end of the study period (OR 1.32 95% CI: 1.18–1.49, *P* < 0.001). Overall, 2.3% of all organisms grown were *Acanthamoeba* sp. (n = 35/1539). The corneal scrape samples were less likely to be positive for *Acanthamoeba* sp. in spring (OR 0.26 95% CI: 0.08–0.69, *P* = 0.011) or summer (OR 0.39, CI: 0.15–0.92, *P* = 0.037; Table 1).

## DISCUSSION

This study has identified several seasonal and temperature trends related to MK in the northwest of England. The most commonly isolated pathogen from our 12-year series was coagulase negative staphylococcus,^11^ in keeping with other reports of causative organisms in MK in other regions of the UK.^10,15,16^ Although we detected a temporal trend, with a slight reduction in gram-positive organisms being isolated toward the end of the study, further studies are required to ascertain the reasons for this finding. An increasing trend in contact lens wear over the period of study^17^ may have influenced the pattern of microbial pathogens isolated from corneal ulcers over time, away from gram-positive organisms (more often associated with ocular surface disease or prior ocular surgery)^1^ toward contact lens-related pathogens (ie gram-negatives or *Acanthamoeba* sp.).^11^ Also, increasing culture-positivity in summer months over the time period of the study may reflect seasonal trends in contact lens wear in the UK—further research is required to ascertain the impact of patients' contact lens use on seasonality of MK in the UK.

We found that gram-positive organisms were more likely to be present in corneal scrape samples obtained in warmer temperatures, although this is not specifically associated with any season. Others have also reported a similar association between gram-positive organisms (especially *Staphylococcus aureus*) isolated from blood cultures in septicaemia and increase in environmental temperature.^18^ Coagulase-negative staphylococcal cultures were more abundant in autumn months but were not specifically associated with temperature; this implies a role for factors other than temperature in influencing the seasonal pattern, for example humidity/rainfall during autumn or patient-related factors (ie increased foreign travel or contact lens wear) during this season. We also found that gram-negative organisms were more likely to occur during summer, and *Pseudomonas* isolates in particular were more likely to occur in warmer temperatures. This finding has also been observed in other studies of MK in Australia and New York, USA.^7,19^ Strains of *P. aeruginosa* isolated from bacterial keratitis share genotypic similarities with environmental strains that reside in surface water,^20^ and *P. aeruginosa* is more frequently isolated from surface water in summer rather than winter.^21^ Hence, patients exposed to standing water in summer, for example in swimming pools,^21^ may be more likely to develop *Pseudomonas* keratitis in summer months. Nonocular gram-negative infections also exhibit this pattern, with data from the USA and other areas suggesting that gram-negative bloodstream infections are more common in warmer, summer periods.^18,22^ The underlying mechanism remains unknown although a rise in temperature may aid the pathogenicity of gram-negative bacteria through increased production of virulence factors such as lipid A in the lipopolysaccharide capsule.^18^ Conversely, the gram-negative species *Moraxella* was associated with lower temperatures in our study. Previous reports of *Moraxella* sp. in respiratory infections and otitis media have similar findings with more infections during lower temperatures in winter months.^23,24^ One reason for this has been attributed to the increased expression of adhesion molecules by *Moraxella* sp. in colder conditions, allowing better adherence to respiratory epithelial cells and inducing the production of epithelial proinflammatory cytokines (eg IL8) once adherence had occurred.^25^

Fungal isolates were also more likely to occur at lower temperatures, and *Candida* sp. was the most common fungal isolate in this study. *Candida* keratitis is more likely to occur in cooler climates than in tropical regions,^26^ and invasive infections associated with *Candida* sp. occur more frequently in winter months.^27^ Although *Fusarium* sp. is often the most common fungus to be isolated in tropical regions,^26,28^ this was not the case in our patient cohort. Humidity may also play a role in the growth of certain fungi,^29,30^ although we did not investigate the effect of humidity as part of this study. Seasonal variations in host vitamin D3 levels may also have an impact on the host response to fungal pathogens at the ocular surface. In vitro studies have shown that higher vitamin D3 levels (which may occur naturally in summer months) are associated with an anti-inflammatory effect, with reduced gene expression of *TLR2*, *TLR4*, and *Dectin-1* pattern-recognition receptors as well as *IL17* and *IFNG* in peripheral blood mononuclear cells in response to *Candida albicans*.^31^ Further studies are required to explore such a seasonal host–pathogen response in MK.

Our analysis found *Acanthamoeba* sp. to be more prevalent in corneal scrape samples obtained in winter months. This is in contrast to previous reports of higher incidence of *Acanthamoeba* keratitis in summer rather than winter in other temperate regions, that is Portland, OR, USA, and in Toronto, ON, Canada,^32,33^ as well as increased isolation of free-living *Acanthamoeba* both in surface water and in domestic water tanks during summer months.^34,35^ One possible reason for our observation of increased *Acanthamoeba* cases, and in fact increased culture-positivity overall, in winter may be that a higher proportion of patients had travelled to warmer climates for vacation during winter and returned to Manchester having contracted ocular infection in another country. Other studies have shown that clinical isolates of *Acanthamoeba castellani* grow optimally at cooler temperatures (ie 32°C),^36^ rather than at the temperature of the normal corneal surface (ie approximately 32°C–34°C),^37,38^ and that higher temperatures over 37°C induce cyst formation.^39^ Lower temperatures in winter may play a role in cooling the corneal surface,^38^ thereby theoretically increasing the risk of infection with *Acanthamoeba* sp. Overall, we found that some organisms, for example *Acanthamoeba*, *Moraxella*, and fungi showed an increase in numbers isolated over the study period. Possible reasons for this may include an increased referral of cases to our tertiary referral center over time, increase in monthly disposable contact lens use in patients in our region, or in fact a true increase in the incidence of these pathogens as causes of keratitis over time. Although we did not detect a direct increase in *Acanthamoeba* keratitis or *Fusarium* keratitis in association with the global outbreaks of these diseases due to contaminated contact lens solutions, we and others have detected an increase in *Acanthamoeba* and fungal keratitis over time even following the resolution of these outbreaks.^40,41^

With regard to the strengths and limitations of this study, a major strength is that through the use of such a large dataset, we were able to take into account temporal trends during the 12-year study period. This study was conducted in a tertiary referral center, and so there is a possibility that a greater proportion of cases may have been included with more severe keratitis compared with other centers. As such, some patients may already have commenced antimicrobial treatment, initiated elsewhere, prior to presentation. However, we included in this study all corneal scrape specimens taken during the study period, rather than limiting to contact lens-related MK alone. Although we have focused on culture-positive samples, corneal scrapes are not always taken for every case of MK and as such we may have underestimated the microbiological burden of disease in this retrospective study. Our study was based in predominantly a large urban area, and we have used monthly average temperatures from the Met Office temperature measurement stations nearest to Manchester Royal Eye Hospital. There is a possibility that “urban microclimates” may exist within the city environment in which buildings can absorb and reflect heat, thus increasing temperatures and affecting local wind patterns, which may have an impact on the microbiological profile within this environment.^42^ Some of the infections recorded in this study may have been acquired during patients' travel overseas and so may not be affected by seasonal temperature fluctuations in Manchester. Future studies could take patients' travel history into account to provide more information on the origin of any organism cultured from corneal scrapes. In addition, further research is required to associate other meteorological data, such as rainfall and humidity, with seasonal peaks in organisms cultured from keratitis samples in the UK. As previous studies have shown, many pathogens that cause keratitis have a predilection for specific environmental niches, for example surface water in summer months for *Pseudomonas* sp.; hence further research into environmental factors that influence these pathogens may increase the understanding of disease pathogenesis.

Since limited data exist on seasonal trends in MK around the world, the data we have provided in this study aim to allow a greater understanding of the effect of temperature and season on the prevalence of causative organisms in MK in the UK. This may aid clinicians in the future planning of regional antimicrobial treatment strategies.

## References

[1] Bourcier T Thomas F Borderie V Bacterial keratitis: predisposing factors, clinical and microbiological review of 300 cases. Br J Ophthalmol. 2003;87:834–838. 12812878 10.1136/bjo.87.7.834 PMC1771775

[2] Dart JK Stapleton F Minassian D Contact lenses and other risk factors in microbial keratitis. Lancet. 1991;338:650–653. 1679472 10.1016/0140-6736(91)91231-i

[3] Keay L Edwards K Naduvilath T Microbial keratitis predisposing factors and morbidity. Ophthalmology. 2006;113:109–116. 16360210 10.1016/j.ophtha.2005.08.013

[4] Seal DV Kirkness CM Bennett HG Population-based cohort study of microbial keratitis in Scotland: incidence and features. Cont Lens Anterior Eye. 1999;22:49–57. 16303406 10.1016/s1367-0484(99)80003-4

[5] Ni N Nam EM Hammersmith KM Seasonal, geographic, and antimicrobial resistance patterns in microbial keratitis: 4-year experience in eastern Pennsylvania. Cornea. 2015;34:296–302. 25603231 10.1097/ICO.0000000000000352

[6] Stapleton F Keay LJ Sanfilippo PG Relationship between climate, disease severity, and causative organism for contact lens-associated microbial keratitis in Australia. Am J Ophthalmol. 2007;144:690–698. 17727808 10.1016/j.ajo.2007.06.037

[7] Green M Apel A Stapleton F A longitudinal study of trends in keratitis in Australia. Cornea. 2008;27:33–39. 18245964 10.1097/ICO.0b013e318156cb1f

[8] Otri AM Fares U Al-Aqaba MA Profile of sight-threatening infectious keratitis: a prospective study. Acta Ophthalmol. 2013;91:643–651. 22863376 10.1111/j.1755-3768.2012.02489.x

[9] Ibrahim YW Boase DL Cree IA Incidence of infectious corneal ulcers, portsmouth study, UK. J Clin Exp Ophthamol. 2012;S6:001.

[10] Ibrahim YW Boase DL Cree IA Epidemiological characteristics, predisposing factors and microbiological profiles of infectious corneal ulcers: the Portsmouth corneal ulcer study. Br J Ophthalmol. 2009;93:1319–1324. 19502241 10.1136/bjo.2008.151167

[11] Tan SZ Walkden A Au L Twelve-year analysis of microbial keratitis trends at a UK tertiary hospital. Eye (Lond). 2017;31:1229–1236. 28452995 10.1038/eye.2017.55 PMC5584503

[12] Cilloniz C Ewig S Gabarrus A Seasonality of pathogens causing community-acquired pneumonia. Respirology. 2017;22:778–785. 28093834 10.1111/resp.12978

[13] R Core Team. R: A Language and Environment for Statistical Computing. Vienna, Austria: R Foundation for Statistical Computing; 2013.

[14] Lesnoff M Lancelot R aod: analysis of overdispersed data. R package version 1.3. 2012.

[15] Orlans HO Hornby SJ Bowler IC In vitro antibiotic susceptibility patterns of bacterial keratitis isolates in Oxford, UK: a 10-year review. Eye (Lond). 2011;25:489–493. 21252952 10.1038/eye.2010.231 PMC3171240

[16] Kaye S Tuft S Neal T Bacterial susceptibility to topical antimicrobials and clinical outcome in bacterial keratitis. Invest Ophthalmol Vis Sci. 2010;51:362–368. 19684005 10.1167/iovs.09-3933

[17] Morgan PB Efron N Helland M Global trends in prescribing contact lenses for extended wear. Cont Lens Anterior Eye. 2011;34:32–35. 20630794 10.1016/j.clae.2010.06.007

[18] Eber MR Shardell M Schweizer ML Seasonal and temperature-associated increases in gram-negative bacterial bloodstream infections among hospitalized patients. PLoS One. 2011;6:e25298. 21966489 10.1371/journal.pone.0025298 PMC3180381

[19] Gorski M Genis A Yushvayev S Seasonal variation in the presentation of infectious keratitis. Eye Contact Lens. 2016;42:295–297. 26618904 10.1097/ICL.0000000000000213

[20] Shankar J Sueke H Wiehlmann L Genotypic analysis of UK keratitis-associated pseudomonas aeruginosa suggests adaptation to environmental water as a key component in the development of eye infections. FEMS Microbiol Lett. 2012;334:79–86. 22708785 10.1111/j.1574-6968.2012.02621.x

[21] Barben J Hafen G Schmid J Pseudomonas aeruginosa in public swimming pools and bathroom water of patients with cystic fibrosis. J Cyst Fibros. 2005;4:227–231. 16081326 10.1016/j.jcf.2005.06.003

[22] Perencevich EN McGregor JC Shardell M Summer peaks in the incidences of gram-negative bacterial infection among hospitalized patients. Infect Control Hosp Epidemiol. 2008;29:1124–1131. 19031546 10.1086/592698

[23] Anita KB Faseela TS Yashvanth KR Moraxella catarrhalis: an often overlooked pathogen of the respiratory tract. J Clin Diagn Res. 2011;5:495–497.

[24] Gisselsson-Solen M Henriksson G Hermansson A Risk factors for carriage of AOM pathogens during the first 3 years of life in children with early onset of acute otitis media. Acta Otolaryngol. 2014;134:684–690. 24834935 10.3109/00016489.2014.890291

[25] Spaniol V Troller R Aebi C Physiologic cold shock increases adherence of Moraxella catarrhalis to and secretion of interleukin 8 in human upper respiratory tract epithelial cells. J Infect Dis. 2009;200:1593–1601. 19835476 10.1086/644640

[26] Leck AK Thomas PA Hagan M Aetiology of suppurative corneal ulcers in Ghana and south India, and epidemiology of fungal keratitis. Br J Ophthalmol. 2002;86:1211–1215. 12386069 10.1136/bjo.86.11.1211 PMC1771352

[27] Edi-Osagie NE Emmerson AJ Seasonality of invasive Candida infection in neonates. Acta Paediatr. 2005;94:72–74. 15858964 10.1111/j.1651-2227.2005.tb01791.x

[28] Chang CW Ho CK Chen ZC Fungi genus and concentration in the air of onion fields and their opportunistic action related to mycotic keratitis. Arch Environ Health. 2002;57:349–354. 12530603 10.1080/00039890209601420

[29] Thomas PA Fungal infections of the cornea. Eye (Lond). 2003;17:852–862. 14631389 10.1038/sj.eye.6700557

[30] Kredics L Narendran V Shobana CS Filamentous fungal infections of the cornea: a global overview of epidemiology and drug sensitivity. Mycoses. 2015;58:243–260. 25728367 10.1111/myc.12306

[31] Khoo AL Chai LY Koenen HJ 1,25-dihydroxyvitamin D3 modulates cytokine production induced by Candida albicans: impact of seasonal variation of immune responses. J Infect Dis. 2011;203:122–130. 21148505 10.1093/infdis/jiq008 PMC3086448

[32] McAllum P Bahar I Kaiserman I Temporal and seasonal trends in Acanthamoeba keratitis. Cornea. 2009;28:7–10. 19092396 10.1097/ICO.0b013e318181a863

[33] Page MA Mathers WD Acanthamoeba keratitis: a 12-year experience covering a wide spectrum of presentations, diagnoses, and outcomes. J Ophthalmol. 2013;2013:670242. 23840938 10.1155/2013/670242 PMC3694549

[34] Mathers WD Sutphin JE Lane JA Correlation between surface water contamination with amoeba and the onset of symptoms and diagnosis of amoeba-like keratitis. Br J Ophthalmol. 1998;82:1143–1146. 9924301 10.1136/bjo.82.10.1143 PMC1722385

[35] Taravaud A Ali M Lafosse B Enrichment of free-living amoebae in biofilms developed at upper water levels in drinking water storage towers: an inter- and intra-seasonal study. Sci Total Environ. 2018;633:157–166. 29573682 10.1016/j.scitotenv.2018.03.178

[36] Nielsen MK Nielsen K Hjortdal J Temperature limitation may explain the containment of the trophozoites in the cornea during Acanthamoeba castellanii keratitis. Parasitol Res. 2014;113:4349–4353. 25204727 10.1007/s00436-014-4109-0

[37] Lorenzo-Morales J Khan NA Walochnik J An update on acanthamoeba keratitis: diagnosis, pathogenesis and treatment. Parasite. 2015;22:10. 25687209 10.1051/parasite/2015010 PMC4330640

[38] Slettedal JK Ringvold A Correlation between corneal and ambient temperature with particular focus on polar conditions. Acta Ophthalmol. 2015;93:422–426. 25571763 10.1111/aos.12657

[39] Aqeel Y Siddiqui R Iftikhar H The effect of different environmental conditions on the encystation of Acanthamoeba castellanii belonging to the T4 genotype. Exp Parasitol. 2013;135:30–35. 23769934 10.1016/j.exppara.2013.05.017

[40] Cheung N Nagra P Hammersmith K Emerging trends in contact lens-related infections. Curr Opin Ophthalmol. 2016;27:327–332. 27176217 10.1097/ICU.0000000000000280

[41] Farrell S McElnea E Moran S Fungal keratitis in the republic of Ireland. Eye (Lond). 2017;31:1427–1434. 28524886 10.1038/eye.2017.82 PMC5639195

[42] Met Office. National Meteorological Library and Archive Fact Sheet 14: Microclimates (Version 01). London: The Met Office; 2016.

